# Evaluation of the therapeutic potential of cerebrolysin and/or lithium in the male Wistar rat model of Parkinson’s disease induced by reserpine

**DOI:** 10.1007/s11011-023-01189-4

**Published:** 2023-02-27

**Authors:** Engy K. Tharwat, Ahmed O. Abdelaty, Alaa I. Abdelrahman, Hebatallah Elsaeed, Ayatallah Elgohary, Amena S. El-Feky, Yasmina M. Ebrahim, Alaa Sakraan, Hossam A. Ismail, Yasser A. Khadrawy, Heba S. Aboul Ezz, Neveen A. Noor, Heba M. Fahmy, Haitham S. Mohammed, Faten F. Mohammed, Nasr M. Radwan, Nawal A. Ahmed

**Affiliations:** 1 Biotechnology Department, Faculty of Science, Cairo University, Cairo, Egypt; 2 Zoology Department, Faculty of Science, Cairo University, Cairo, Egypt; 3 Faculty of Medicine, Cairo University, Cairo, Egypt; 4 School of Biotechnology, Badr University in Cairo, Badr City, Cairo Egypt; 5 Department of Pharmacology and Toxicology, Faculty of Pharmacy, Cairo University, Cairo, Egypt; 6 Biophysics Department, Faculty of Science, Tanta University, Tanta, Egypt; 7 Medical Physiology Department, Medical Division, National Research Center, Dokki, Egypt; 8 Biophysics Department, Faculty of Science, Cairo University, Cairo, Egypt; 9 Faculty of Veterinary Medicine, Cairo University, Cairo, Egypt; 10 Department of Zoology, Faculty of Science, Cairo University, Giza, Egypt

**Keywords:** Parkinson’s disease, Cerebrolysin, Lithium, Monoamines, Oxidative stress, Inflammation

## Abstract

Parkinson’s disease (PD) is the second most prevalent neurodegenerative disease worldwide and represents a challenge for clinicians. The present study aims to investigate the effects of cerebrolysin and/or lithium on the behavioral, neurochemical and histopathological alterations induced by reserpine as a model of PD. The rats were divided into control and reserpine-induced PD model groups. The model animals were further divided into four subgroups: rat PD model, rat PD model treated with cerebrolysin, rat PD model treated with lithium and rat PD model treated with a combination of cerebrolysin and lithium. Treatment with cerebrolysin and/or lithium ameliorated most of the alterations in oxidative stress parameters, acetylcholinesterase and monoamines in the striatum and midbrain of reserpine-induced PD model. It also ameliorated the changes in nuclear factor-kappa and improved the histopathological picture induced by reserpine. It could be suggested that cerebrolysin and/or lithium showed promising therapeutic potential against the variations induced in the reserpine model of PD. However, the ameliorating effects of lithium on the neurochemical, histopathological and behavioral alterations induced by reserpine were more prominent than those of cerebrolysin alone or combined with lithium. It can be concluded that the antioxidant and anti-inflammatory effects of both drugs played a significant role in their therapeutic potency.

## Introduction

Parkinson’s disease (PD) stands as the second most prevalent progressive neurodegenerative disorder and the most common movement disorder, causing morbidity and mortality and imposing a major health burden (Kowal et al. 2013). It involves death and reduction of dopaminergic neurons in substantia nigra pars compacta and striatum sequentially (Caggiu et al. 2019). Individuals suffering from Parkinson’s disease show severe motor deficits including tremors, bradykinesia, postural instability and rigidity (Neil and David 2008). Previous studies have focused on the striatum, since dopamine depletion in the striatum has been established as the main cause of PD (Huang et al. 2019). Most notably, the midbrain was identified as one of the principle brain regions exhibiting metabolic changes in PD rats (Yang et al. 2020).

Reserpine-induced PD animal model reflects numerous features of PD neuropathology and mimics human PD (Nade et al. 2015; Chaudhary 2020). Reserpine is a powerful inhibitor of vesicular monoamine transporter 2 (VMAT2) inducing tremors, muscular rigidity, and akinesia, by depleting central monoamine stores (Nade et al. 2015; Vandana et al. 2015; Rijntjes and Meyer, 2019), in particular in motor function centers (Fernandes et al. 2012).

Recently, Li et al. (2022) demonstrated that reserpine induced motor and non-motor symptoms similar to PD. The authors found that reserpine caused alpha- synuclein deposition, autophagy impairment and tyrosine hydroxylase reduction in the substantia nigra and proposed reserpine model as a valuable tool in PD studies.

Cerebrolysin is a peptide extracted from porcine brain. Its low-molecular biologically active peptide content (25%) enables it to pass through the blood-brain barrier (Hartbauer et al. 2001). Several studies demonstrated the neuroprotective properties of cerebrolysin which underlie its efficiency in treating neurodegenerative diseases such as schizophrenia (Flores and Atzori 2014), vascular dementia (Plosker and Gauthier 2009), traumatic brain injury (Alzoubi et al. 2018), stroke (Zhang et al. 2013), Alzheimer’s disease (Ubhi et al. 2013), and Parkinson’s disease (Nasrolahi et al. 2018).

The neurotrophic effects of cerebrolysin are mediated by its ability to prevent excitotoxicity, free radical formation, and inflammatory responses (Nasrolahi et al. 2018; Moghazy et al. 2019; El-Marasy et al. 2021). The beneficial pharmacological actions underlying its clinical efficacy include enhancement of synaptic regeneration (Rockenstein et al. 2003), promotion of neuronal survival (Safarova et al. 2002) and augmentation of progenitor cell migration and neurogenesis (Zhang et al. 2010; Abdel-Salam et al. 2014). The promising therapeutic effect of cerebrolysin in rat models of PD induced by 6-hydroxydopamine (Noor et al. 2016) and rotenone (Abdel-Salam et al. 2014) and preclinical rat model of PD (Requejo et al. 2018) has been previously investigated.

The neuroprotective properties of lithium are well documented (Diniz et al. 2013; Motaghinejad et al. 2016) and support its use as a potential therapeutic drug in treating neurodegenerative diseases. The reported mechanisms of action of lithium involve the activation of neurotrophic factors and the reduction of oxidative stress, apoptosis and neuro-inflammation (Li et al. 2016; Khan et al. 2017). It has also been shown to enhance neuroprotective cellular cascades, reduce excitotoxicity and maintain mitochondrial stability (Lazzara and Kim 2015). Several studies demonstrated the neuroprotective effect of lithium in animal models of PD (Guttuso et al. 2019; Wen et al. 2019; Gromova et al. 2015) has found that there is a pharmacokinetic synergism between lithium and cerebrolysin which enhances lithium accumulation in brain tissues.

The aim of the present study is to investigate the therapeutic potential of cerebrolysin and lithium against Parkinson’s disease using a rat model induced by reserpine. This study extends to evaluate whether the combination of the two drugs would be beneficial in improving the neurochemical, behavioral and histopathological alterations induced in the present PD model.

## Materials and methods

### Animals

The present experiment was performed with fifty male Wistar rats. Their weights ranged from 150 to 250 g. The animals were obtained from a local supplier and left for one week to acclimatize with the animal house conditions. Every five rats were housed in a cage and provided with rat chow and water ad libitum. All rats were maintained under 12 h light/12-hour dark conditions, controlled temperature (25 ± 2◦ C), and airflow. All procedures to reduce animal pain and suffering were performed under the International Guidelines for Care and Use of Laboratory Animals and ethical approval was taken from the Institutional Animal Care and Use Committee (CU-IACUC) of the Faculty of Science, Cairo University (Registration no.: CU I F 17 21).

### Drugs and chemicals

Reserpine (Mallinckrodlt. Inc, Paris-Kantucky) was dissolved in glacial acetic acid (1 µg/µl) and completed with distilled water to reach 25 ml. Cerebrolysin was purchased from EVER Neuropharma GmbH A-4866 Unterach, Austria. Lithium chloride was obtained from BDH (England). Ethylene diaminetetraacetic acid (EDTA), thiobarbituric acid (TBA), trichloroacetic acid (TCA), acetylthiocholine iodide, 5,5’-dithiobis-(2-nitrobenzoic acid) (DTNB), and 96% ethanol, N-(1-Naphthyl) ethylenediaminedihydrochloride (NED), phosphoric acid, sulfonamide, and perchloric acid were supplied by Sigma Aldrich. Norepinephrine bitartrate salt (Sigma, Taufkirchen – Germany), dopamine hydrochloride and serotonin (Fluka – Sigma – Aldrich, Taufkirchen – Germany) were used to prepare the standards. n-heptane (Labscan Ltd., Dublin, Ireland), 1-butanol (POCH SA, Gliwice, Poland), hydrochloric acid, acetic acid, ethyl alcohol (EDWIC, El-Nasr Pharmaceutical Chemicals Co., Egypt), sodium acetate (Fluka, Buchs, Switzerland), Iodine (Panreac, Barcelona Spain), ethylenediaminetetraacetic acid (EDTA) (S.D. fine – Chem Ltd. Mumbai, India), sodium sulfite and O-phthalaldehyde (OPT) (Merck, Schuchardt, Germany) were used for the quantitative determination of monoamines in the striatum and midbrain.

### Experimental design

Rats were divided into 5 groups ten animals each. The control group was group (1) and received saline for 6 weeks. Group (2) was the reserpine-induced model of Parkinson’s disease in which rats received a daily intraperitoneal (i.p.) injection of reserpine (0.2 mg/kg) for 6 weeks (Fernandes et al. 2012). Group (3) represented the reserpinized rats treated with cerebrolysin (2.5 ml/kg) (Noor et al. 2016) in which rats were injected intraperitoneally with reserpine for 3 weeks and then with both reserpine and cerebrolysin intraperitoneally for 3 weeks with 1 h interval between the two injections. Group (4) was the reserpinized rats treated with lithium (25 mg/kg) according to Ostrova et al. (2019). The rats received an i.p. injection of resepine for 3 weeks then an i.p. injection of reserpine and an oral administration of lithium separated by 1 h in between for 3 weeks. Group (5) represented the reserpinized rats treated with the combination of cerebrolysin and lithium. In these rats, a daily i.p. injection of reserpine was given for 3 weeks then a daily i.p. injection of reserpine and cerebrolysin followed by a daily oral administration of lithium for 3 weeks with a 1-hour gap between every 2 injections.

### Behavioral test

#### Grip strength test

The grip strength test was carried out as described by Marcioli et al. (2012). It is a measure of neuromuscular function and neurotoxicity and is used to assess maximal muscle strength of combination between forelimbs and hindlimbs by grasping of rats in a grid-connected to a sensor. Every rat was rested in a grid from forepaws only and the grid was 2 cm above the platform. Rats were held by the tail and forced to grasp a rigid bar attached to a digital force gauge (Aikoh Engineering Corporation, Osaka, Japan). The rat was held from the tail to allow it to grasp on the grid and the value in the grip strength meter was recorded. Each rat was gently pulled backward by the tail and before unclasping the bar, the tension reading of the digital force gauge was recorded as grip strength. Each rat performed the test three consecutive times and the average value was calculated as the grip strength force of the rat.

### Handling of tissue samples

The animals were sacrificed by sudden decapitation at the end of the experimental period, 8 animals were used for neurochemical tests and 2 animals for histopathological tests. The brain of each animal was rapidly dissected out, transferred quickly to an ice-cold Petri dish and divided into two equal halves. It was then dissected to obtain the midbrain and striatum. Each sample was weighed and kept at − 58 °C until analyzed. The left half of each brain was homogenized in 5% w/v 20 mM phosphate buffer, pH 7.6, and centrifuged. The supernatant was used for the assay of malondialdehyde (MDA), reduced glutathione (GSH) and nitric oxide (NO) levels. The right half was used to determine monoamine levels.

### Neurochemical analysis

#### Determination of lipid peroxidation

According to the method of Ruiz-Larrea et al. (1994), lipid peroxidation (MDA) was performed in the striatum and midbrain. The absorbance of the red complex produced from the reaction between thiobarbituric acid reactive substances and thiobarbituric acid was read at 532 nm in a Helios Alpha UV-Visible spectrophotometer (ThermoSpectronic, England).

#### Determination of reduced glutathione (GSH)

Ellman’s technique was used to determine GSH (Ellman 1959). The method relies on the reduction of Ellman’s reagent by GSH –SH groups to produce 2-nitro-s-mercaptobenzoic acid, which has a bright yellow hue that can be detected spectrophotometrically at 412 nm.

#### Determination of nitric oxide (NO) level

The level of nitric oxide (NO) was measured as nitrite using the Griess reagent according to Moshage et al. procedure (Moshage et al. 1995). Nitrite is converted to a deep purple azo compound once Griess reagent is added, and its absorbance is measured spectrophotometrically at 450 nm.

#### Determination of acetylcholinesterase activity

The activity of acetylcholinesterase (AChE) was measured using the technique of Gorun et al. (1978) which is a modification of the method of Ellman et al. (1961). The method relies on acetylthiocholine iodide hydrolysis by AChE to form thiocholine which reduces the –SH reagent DTNB yielding thionitrobenzoic acid. The later is a yellow-colored anion whose absorption is read spectrophotometrically at 412 nm.

#### Determination of TNF-α

Tumor necrosis factor-alpha (TNF-alpha) measurement was carried out using rat TNF-alpha Elisa kit Catalog No. SG-20,127 which was obtained from Sino Gene Clon Biotech Co., Ltd. (Hangzhou, China). The color developed was read at 450 nm with a microtiter plate reader. The concentration was then determined from a standard curve.

#### Determination of nuclear factor-kappa (NF-κ)

Nuclear factor kappa B (NF-κ) was assessed by rat NF-κB Elisa kit Catalog No. SG-20,431 purchased from Sino Gene Clon Biotech Co., Ltd. (Hangzhou, China). The absorbance was read against blank at 450 nm with a microtiter plate reader. The level of NF-κ was then obtained from a standard curve.

#### Determination of monoamine concentrations

Monoamine concentrations were measured according to the fluorometric method of Ciarlone (1978). The right half of each brain area was homogenized in ice-cold acidified n-butanol and centrifuged at 2,000 rpm for 5 min. The supernatant of each sample, 2.5 ml, was transferred to a test tube containing 1.6 ml 0.2 N acetic acid and 5 ml heptane. The tubes were vortexed for 30 s and centrifuged at 2,000 rpm for 5 min. 200 µl of the aqueous phase were used for serotonin (5-HT) analysis with o-phthalaldehyde. 1 ml aliquots were transferred into other test tubes for norepinephrine (NE) and dopamine (DA) analysis using iodine. The fluorescence was measured spectrofluorometerically in a model Jasco-FP-6500 (Japan) with a xenon arc lamp 150 W source having an excitation slit band width of 5 nm excitation monochromator and an emission slit band width of 5 nm emission monochromator).

### Histopathological examination

Histological studies were carried out for confirmative/illustrative purposes. At the end of the experiment, the brains were fixed in 10% formaldehyde. Then each brain area was embedded in paraffin, and cut serially at 8 Ǻ sections. Routine staining of sections was performed using hematoxylin–eosin (H&E) and the sections were examined by a light microscope and photographed.

### Statistical analysis

All data were expressed as mean ± standard error of the mean (S.E.M.). Comparisons between the means of different groups of animals and those of control animals were performed using one-way analysis of variance (ANOVA) with the help of SPSS (Statistical Package for Social Sciences, version 14). Significance was set at p < 0.05. When the data were statistically significant, ANOVA test was followed by Duncan as post hoc test.

## Results

### Behavior results

#### Grip strength test

**Fig. 1:**
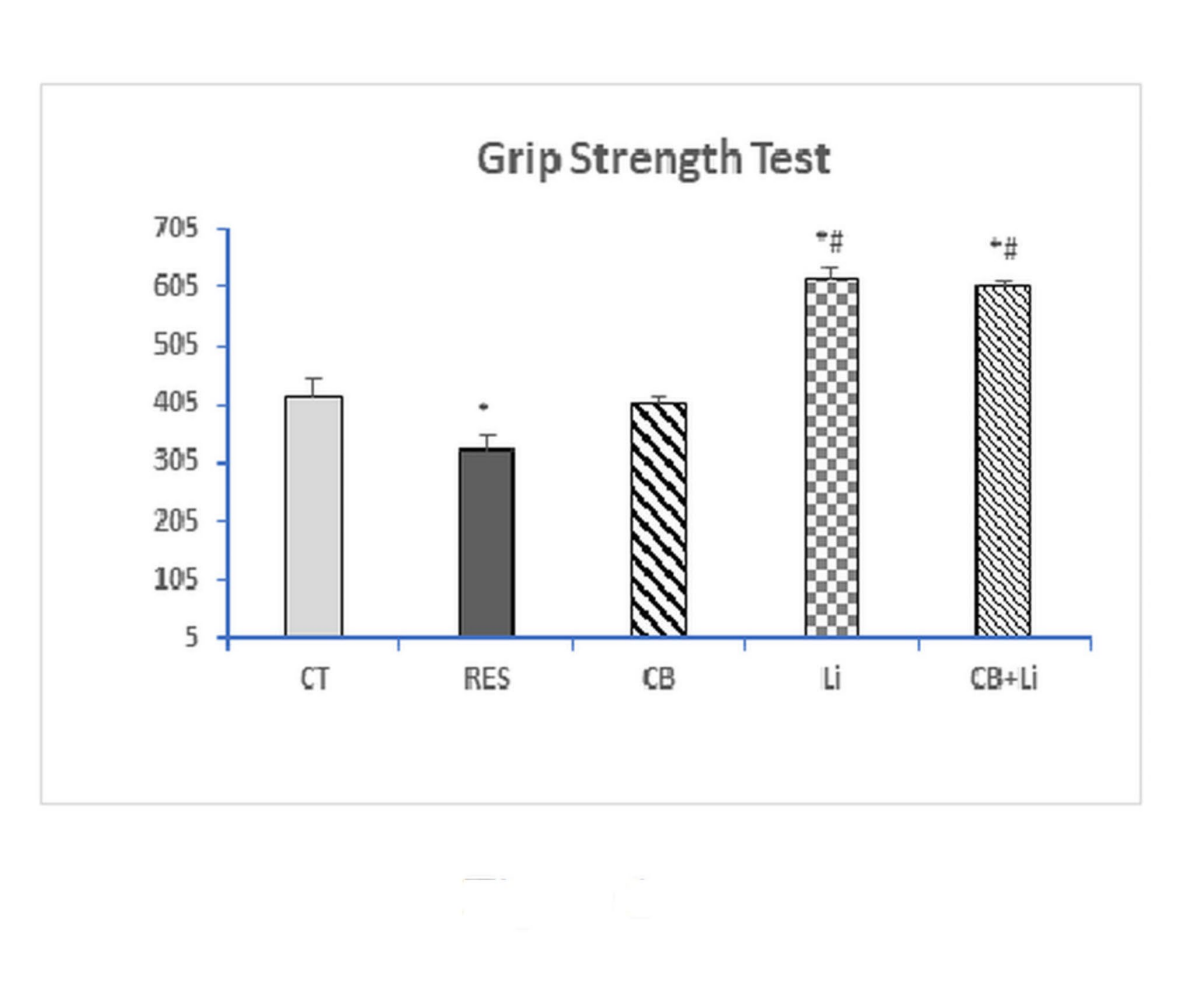
The grip strength test in control (CT) rats, reserpine (RES)-induced PD model, RES-induced model treated with cerebrolysin (CB), RES-induced model treated with lithium (Li), and RES-induced model treated with both CB + Li. *Significant with control (p˂0.05) #Significant with RES model (p˂0.05)

In this test, the latency to grip loss was significantly reduced in the reserpine group recording − 21.82% below the control value (Fig. 1). The i.p. injection of cerebrolysin attenuated the decrease in the grip strength induced by reserpine. Treatment of reserpinized animals with lithium alone or combined with cerebrolysin resulted in a significant increase in the grip strength recording a percentage difference of 48.18% and 45.49%, respectively, when compared to control group.

### Neurochemical results

#### Oxidative stress parameters

**Fig. 2:**
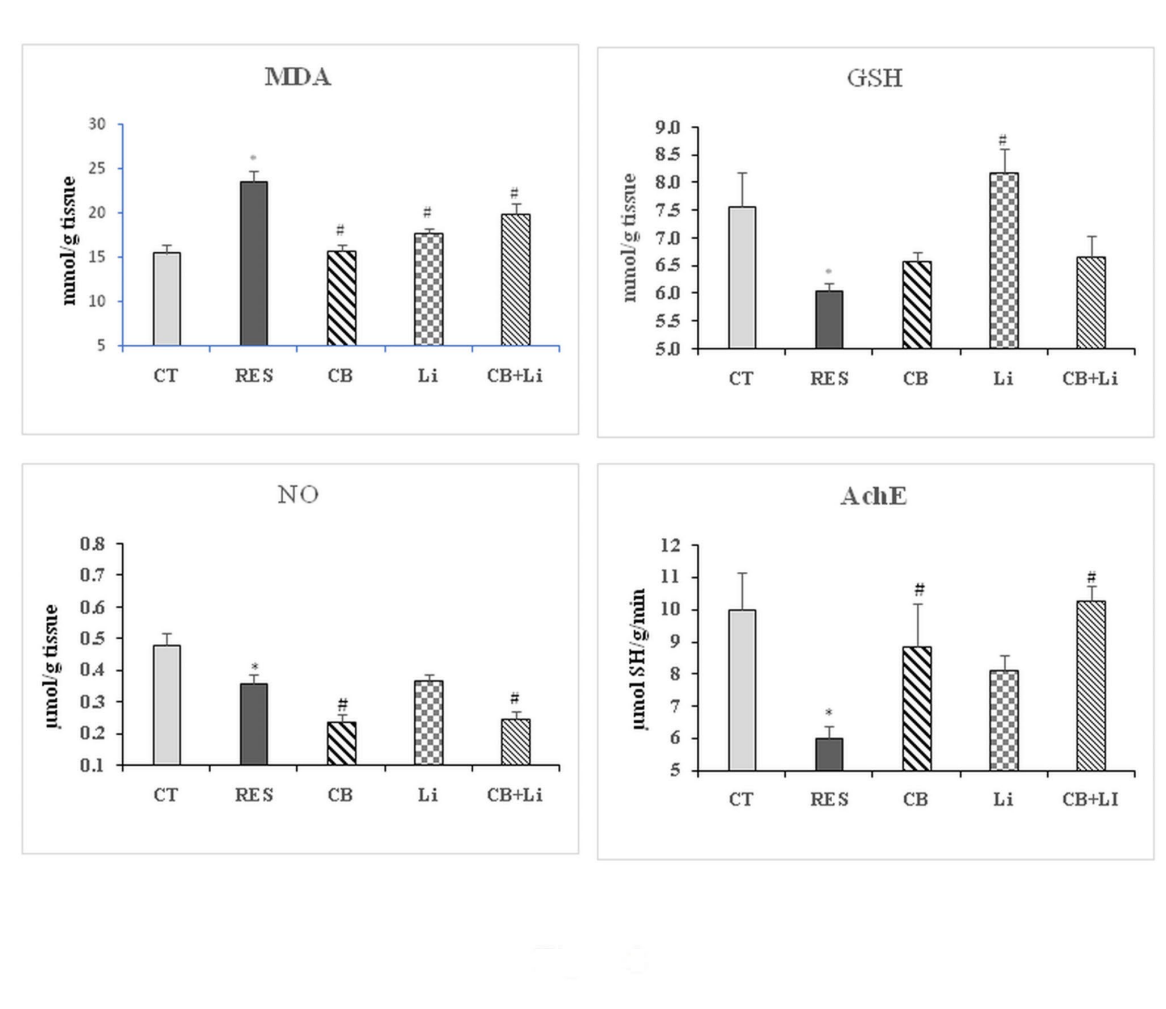
The effect of cerebrolysin, lithium and their combination on the levels of malondialdehyde (MDA), reduced glutathione (GSH), and nitric oxide (NO) and acetylcholinesterase (AChE) activity in the striatum of reserpine-induced model of Parkinson’s disease *Significant with control (p˂0.05) #Significant with RES model (p˂0.05)

In the striatum of the rat model of PD, the i.p. injection of reserpine resulted in a significant increase in MDA levels by 52.42% while the levels of NO and GSH decreased significantly recording − 25.00% and − 20.05% below the control values, respectively (Fig. 2). The i.p. injection of cerebrolysin restored the levels of MDA with an improvement in GSH levels (-13.06%). It also resulted in a further reduction of NO levels to be significant with the control and reserpine model recording − 50.00% below the control levels. The oral administration of lithium attenuated the increase in MDA levels and ameliorated GSH levels. However, there was no change in the levels of NO from the reserpine model levels, the percentage difference from control recording − 22.92%. The treatment of reserpinized animals with the combination of lithium and cerebrolysin improved MDA and GSH levels but reduced NO levels by 47.92% below the control values.

**Fig. 3:**
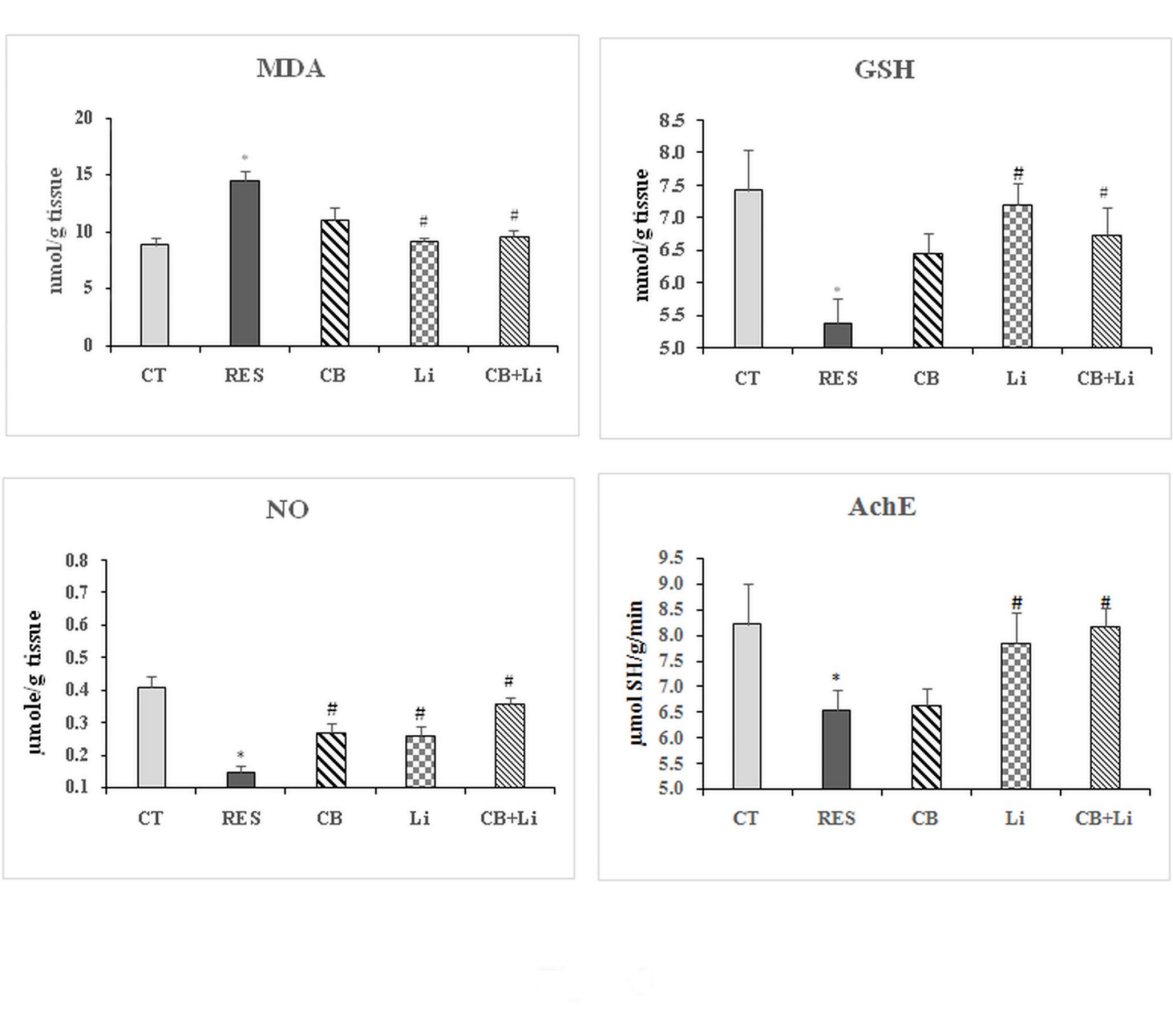
The effect of cerebrolysin, lithium and their combination on the levels of malondialdehyde (MDA), reduced glutathione (GSH), and nitric oxide (NO) and acetylcholinesterase (AChE) activity in the midbrain of reserpine-induced model of Parkinson’s disease *Significant with control (p˂0.05) #Significant with RES model (p˂0.05)

In the midbrain of the rat model of PD injected intraperitoneally with reserpine, a significant increase in the levels of MDA (63.35%) was obtained with a significant decrease in NO (-63.41%) and GSH (-27.59%). The i.p. injection of cerebrolysin improved the levels of MDA, NO, and GSH recording 25.34%, -34.15%, and − 13.06%, respectively, in PD rat model induced by reserpine. The oral administration of lithium restored the levels of MDA and GSH to nearly control values and attenuated the decrease in NO levels being significant with the control and reserpine model. The combined treatment of cerebrolysin and lithium ameliorated the changes in MDA, GSH and NO levels induced by reserpine in the PD rat model (Fig. 3).

#### Acetylcholinesterase activity

Acetylcholinesterase (AChE) activity was decreased significantly by 40.02% in the striatum of reserpine model of PD. Cerebrolysin alone or combined with lithium returned AChE activity to nearly control values while lithium alone attenuated the decrease in the enzyme activity by 19.06% as compared to control values (Fig. 2). Reserpine also decreased the midbrain activity of AChE significantly by 20.58%. The i.p. injection of cerebrolysin failed to restore the activity of AChE. The oral administration of lithium alone improved the enzyme activity while only the combination of the two drugs restored midbrain AChE activity in the reserpine model of PD to control-like values (Fig. 3).

#### Tumor necrosis factor

**Fig. 4:**
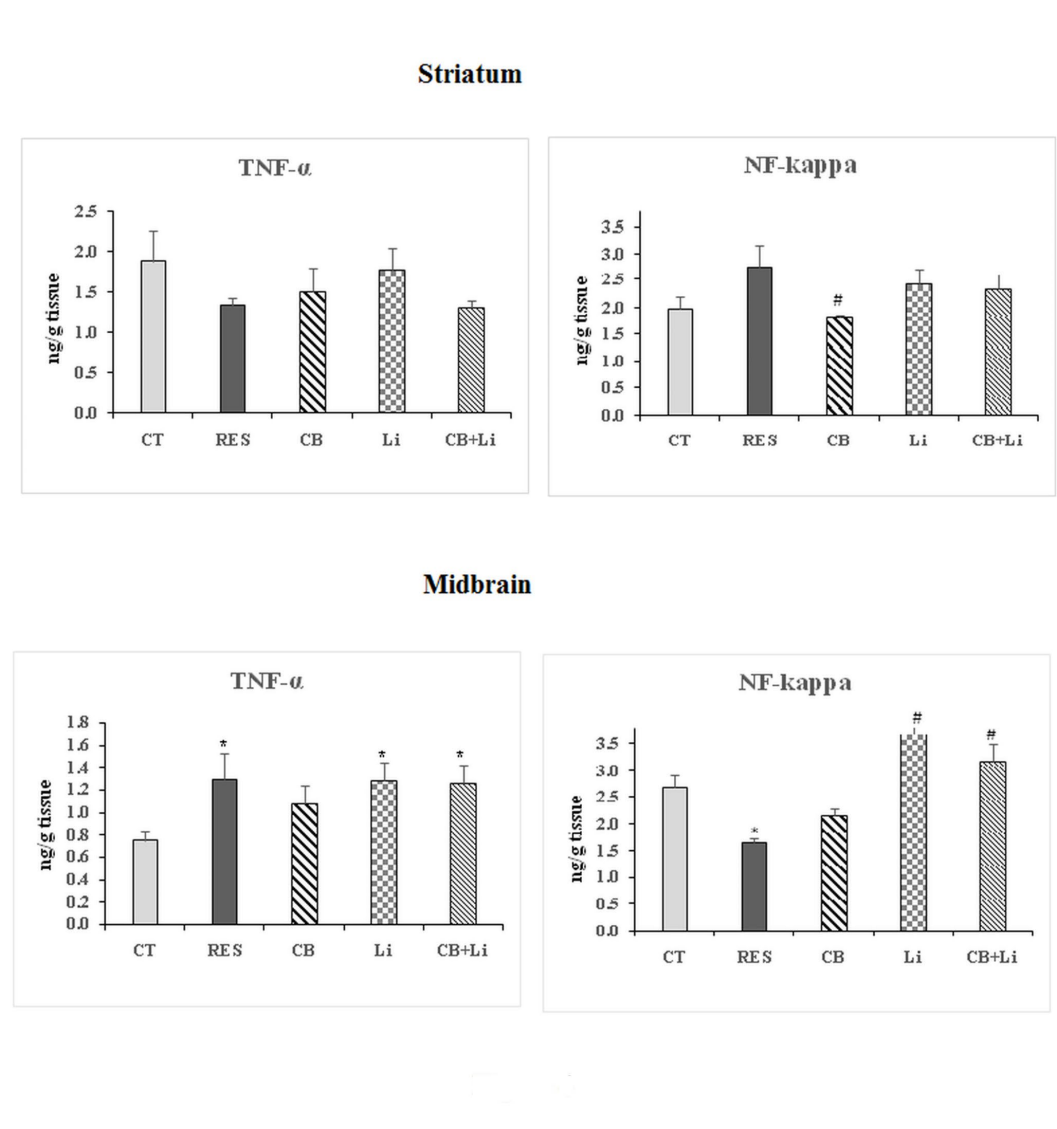
The effect of cerebrolysin, lithium and their combination on the levels oftumor necrosis factor-alpha (TNF-α) and nuclear factor-kappa (NF-kappa) in the striatum and midbrain of reserpine-induced model of Parkinson’s disease *Significant with control (p˂0.05) #Significant with RES model (p˂0.05)

Striatal TNF-α levels were nonsignificantly changed in the three groups and in the reserpine induced model in comparison with control levels. In the mid brain, reserpine showed a significant increase in the levels of TNF-α (73.33%). Cerebrolysin showed an improvement in TNF-α (45.33%). Lithium alone or combined with cerebrolysin increased TNF-α levels significantly above control levels (Fig. 4).

#### Nuclear factor-kappa

In the striatum of the rat model of PD, the i.p. injection of reserpine induced a nonsignificant increase in NF-Kappa. Cerebrolysin reduced NF-kappa levels as compared to the reserpine-induced model. Lithium alone or combined with cerebrolysin showed nonsignificant changes in NF-Kappa (Fig. 4).

In the midbrain, reserpine showed a significant decrease in NF-kappa (-38.52%). Cerebrolysin showed an improvement in NF-kappa (-20.00%). Lithium increased NF-kappa levels recording 37.04% above control levels. The combined treatment of both cerebrolysin and lithium elevated the levels of NF-kappa nonsignificantly by 18.15% as compared with control levels (Fig. 4).

#### Monoamine concentrations

In the striatum of the rat model of PD induced by the i.p. injection of reserpine, a significant decrease in the levels of dopamine (DA), norepinephrine (NE) and serotonin (5-HT) was observed, recording − 30.58%, -36.75% and − 31.87% below the control levels, respectively (Table 1). The oral administration of lithium alone ameliorated this reduction and restored the monoamines to control levels. Treatment with cerebrolysin alone or combined with lithium improved the reduction in the levels of the three monoamines.

**Table 1:** The effect of cerebrolysin, lithium and their combination on the levels of dopamine (DA), norepinephrine (NE) and serotonin (5-HT) in the striatum of reserpine-induced model of Parkinson’s disease

	Control	Reserpine	%D	Cerebrolysin	%D	Lithium	%D	Combination	%D
**DA**	2.91 ± 0.18^**a**^ (7)	2.02 ± 0.10^**b**^ (7)	-30.58	2.58 ± 0.15^**ac**^ (7)	-11.34	2.65 ± 0.11^**ac**^ (7)	-8.93	2.45 ± 0.06^**c**^ (7)	-15.81
**NE**	1.66 ± 0.18^**ac**^ (6)	1.05 ± 0.05^**b**^ (6)	-36.75	1.31 ± 0.09^**bc**^ (6)	-21.08	1.69 ± 0.09^**a**^ (7)	1.81	1.35 ± 0.11^**abc**^ (6)	-18.67
**5-HT**	14.78 ± 1.06^**a**^ (6)	10.07 ± 0.50^**b**^ (6)	-31.87	12.42 ± 0.70^**c**^ (7)	-15.97	14.51 ± 0.54^**a**^ (7)	-1.83	12.33 ± 0.32^**c**^ (6)	-16.58

Values represent mean ± S.E. with the number of animals between parentheses

% D: % difference with respect to control values

Different letters indicate significantly different means at p-value < 0.05

Same letters indicate non significant changes

a: non significant with control

b: significant with control

ab: non significant with control and reserpine model

c: significant with control and reserpine model

A significant decrease in the levels of DA and 5-HT was also recorded in the midbrain of the rat model of PD injected intraperitoneally with reserpine (Table 2). Similar to the striatum, an improvement in DA occurred after treatment of the rat model with cerebrolysin alone or combined with lithium. Cerebrolysin attenuated the decrease in 5-HT levels while lithium treatment restored the levels of both DA and 5-HT to control values. The combination of cerebrolysin and lithium ameliorated the decrease in 5-HT induced by reserpine. Regarding NE, no significant changes from control rats were recorded after treatment of rat PD model with cerebrolysin, lithium or the combination of the two drugs.

**Table 2:** The effect of cerebrolysin, lithium and their combination on the levels of dopamine (DA), norepinephrine (NE) and serotonin (5-HT) in the midbrain of reserpine-induced model of Parkinson’s disease

	Control	Reserpine	%D	Cerebrolysin	%D	Lithium	%D	Combination	%D
**DA**	5.19 ± 0.29^**a**^ (6)	4.14 ± 0.17^**b**^ (6)	-20.23	4.66 ± 0.23^**ab**^ (6)	-10.21	5.11 ± 0.23^**a**^ (7)	-1.54	4.49 ± 0.26^**ab**^ (7)	-13.49
**NE**	1.82 ± 0.11^**ab**^ (6)	1.58 ± 0.06^**a**^ (6)	-13.19	1.86 ± 0.13^**ab**^ (6)	2.20	1.95 ± 0.05^**b**^ (6)	7.14	1.78 ± 0.07^**ab**^ (7)	-2.20
**5-HT**	15.86 ± 0.79^**ac**^ (6)	13.71 ± 0.43^**b**^ (6)	-13.56	14.05 ± 0.47^**bc**^ (6)	-11.41	16.06 ± 0.43^**a**^ (6)	1.26	15.59 ± 0.73^**ac**^ (7)	-1.70

Values represent mean ± S.E. with the number of animals between parentheses

% D: % difference with respect to control values

Different letters indicate significantly different means at p-value < 0.05

Same letters indicate non significant changes

a: non significant with control

b: significant with control

ab: non significant with control and reserpine model

c: significant with control and reserpine model

### The histopathological alterations

The lesions in the striatum were severe in PD model group, the caudate and putamen showing necrosis of neurons (Fig. 3a) with deposition of eosinophilic dense granules in their cytoplasm (Fig. 3b) associated with microgliosis. The internal capsule showed diffuse gliosis (Fig. 4c). The lesions also extended to involve the globus pallidus. Moderate neuronal necrosis of neurons comprising the striatum (Fig.d) was detected with deposition of Lewis body in their cytoplasm (Fig. 4e). However, only gliosis with individual necrosis of neurons occurred in lithium alone or combined with cerebrolysin (Fig. 4f&g).

**Fig. 5:**
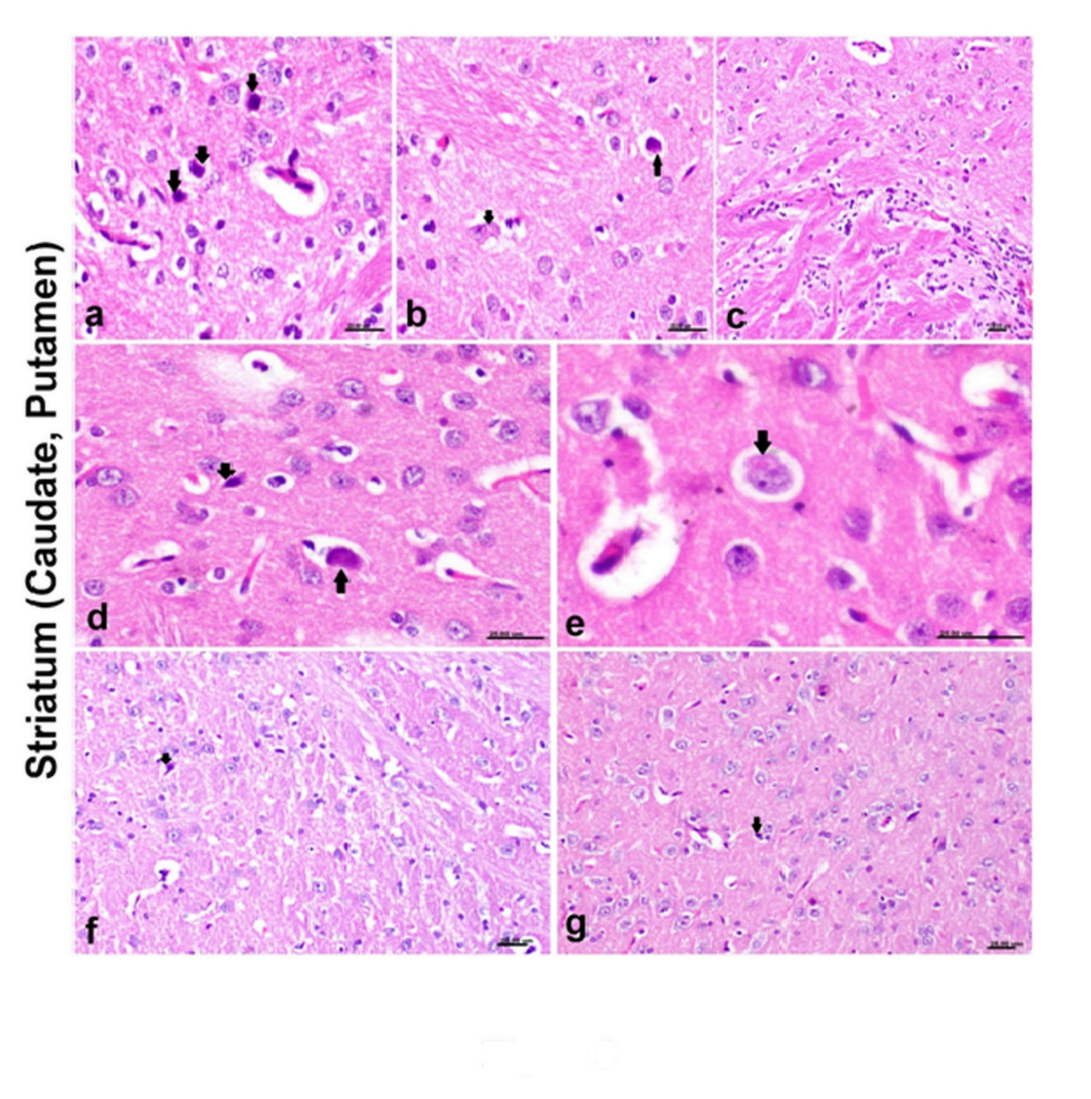
Histological sections of the striatum in different groups. a) PD rat model showing extensive neuronal necrosis (black arrows), (b) PD rat model showing deposition of eosinophilic dense granules in neuronal cytoplasm comprising the caudate (black arrow), c) PD rat model showing marked gliosis of internal capsule, d) cerebrolysin-treated rat showing neuronal necrosis (black arrow), d) cerebrolysin-treated rat showing deposition of eosinophilic Lewis body in neuronal cytoplasm (black arrow), e) lithium-treated rat showing mild glia reaction and individual neuronal degeneration (black arrow), f) lithium + cerebrolysin treated rats also showing mild glia reaction and individual neuronal degeneration (black arrow)

**Fig. 6:**
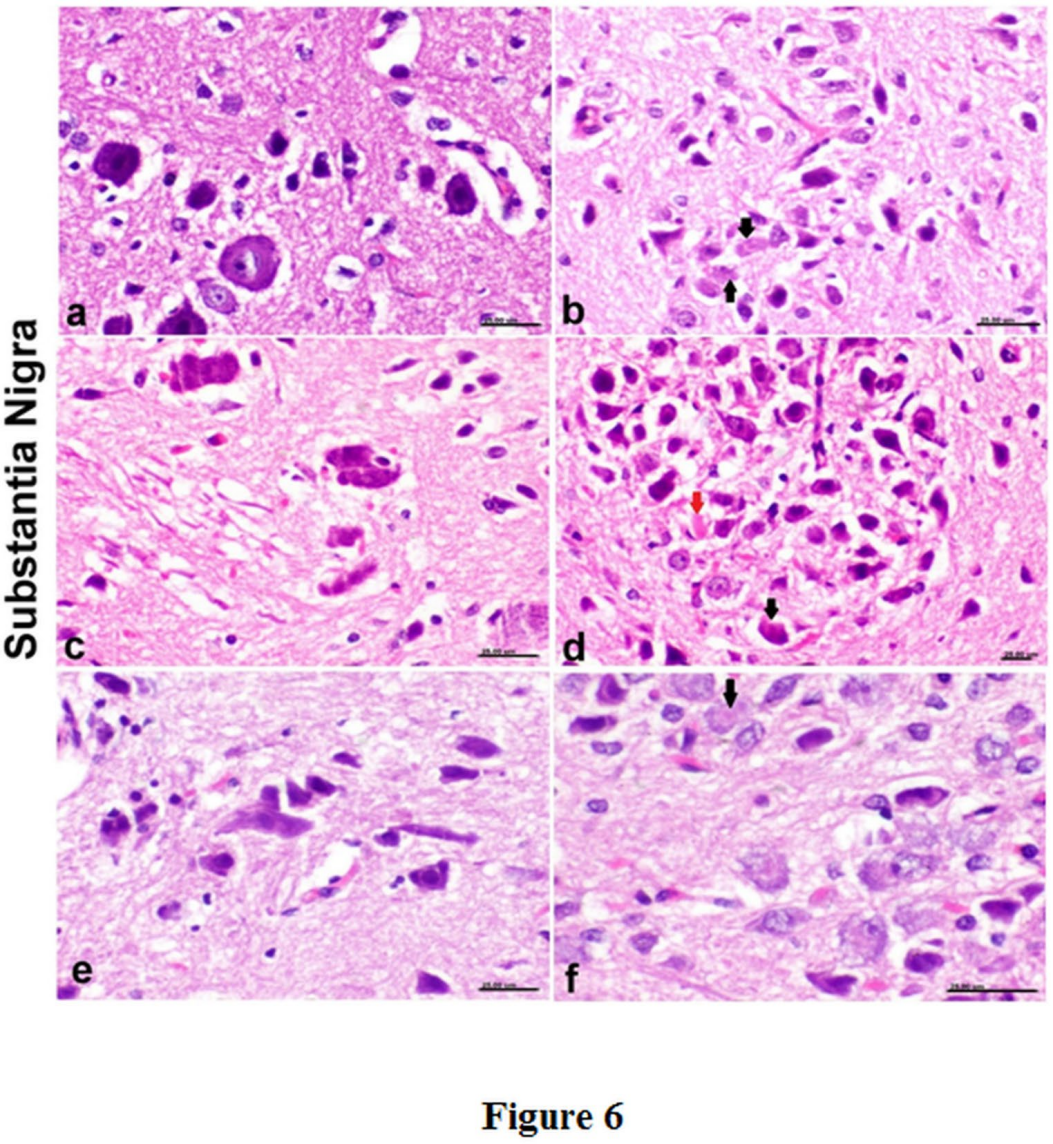
Histological sections of substantia nigra in different groups. a) substantia nigra compacta of PD rat model showing massive loss and destruction, necrosis and shrinkage of dopaminergic neurons, b&c) substantia nigra reticularis of PD rat model showing intracytoplasmic deposition of Lewis body (black arrows) associated with marked loss of neurons (c), d) cerebrolysin-treated rat showing marked necrosis of neurons comprising the substantia nigra reticularis that appeared as dense eosinophilic bodies (red arrows) with intracellular deposition of Lewis body (black arrows), e) lithium-treated rat showing gliosis with neuronal necrosis, f) lithium + cerebrolysin-treated rat showing intracellular deposition of Lewis body (black arrows)

Pronounced neuropathological lesions were encountered in the substantia nigra, the lesions in PD model included both substantia nigra compacta and reticularis. The lesions were characterized by massive loss and destruction, necrosis and shrinkage of dopaminergic neurons with intracytoplasmic and extracellular deposition of Lewis body associated with microgliosis (Fig. 5a-c). The treatment with cerebrolysin did not alleviate the lesions as appearance of degenerated neurons and deposition of Lewis body were detected (Fig. 5d). However, slight reduction in degeneration and shrinkage of dopaminergic neurons with gliosis were detected in the lithium-treated group (Fig. 5e). The glial reaction was decreased in lithium combined with cerebrolysin with reduction in neuronal reaction but deposition of Lewis body was still detected (Fig. 5f) (Fig. 6).

## Discussion

The repeated administration of a low dose of reserpine results in a progressive appearance of motor symptoms in rats which supports the use of reserpine to induce PD animal models (Fernandes et al. 2012; Li et al. 2022). These motor impairments are accompanied by an enhanced oxidative stress which has been implicated as a possible pathophysiological mechanism in PD (Gaki and Papavassiliou 2014; de Farias et al. 2016].

In the present study, the chronic administration of a low dose of reserpine (0.2 mg/kg) for 6 weeks was used to display the pathological features of PD. The present histopathological data revealed severe lesions in the striatum and midbrain of reserpine-induced model with shrinkage and degeneration of most of the dopaminergic neurons in the substantia nigra. In reserpine-induced PD model group, this was reflected behaviorally by a decrease in grip strength that may be attributed to the decreased muscle strength of this group as compared to control group animals (Welch et al. 2013). The present depletion of dopamine (DA) level in the striatum and midbrain of reserpinized animals also supports the development of PD in these animals. It has been reported that the decrease in nigro-striatal dopamine in PD increases the tonic inhibition of the thalamus decreasing motor cortex excitation. This may impair the corticospinal activation of muscle thus reducing muscle strength (David et al. 2012).

Reserpine disrupts the storage of monoamines by inhibiting the vesicular monoamine transporter VMAT2 causing monoamine depletion, transient hypolocomotion and muscle rigidity (Gerlach and Riederer 1996). This inhibition also led to the present depletion of norepinephrine (NE) in the striatum and serotonin (5-HT) in the striatum and midbrain of reserpine-induced PD rats. The depletion of NE and 5-HT may be responsible for PD nonmotor impairments which include sleep disorders (Gómez-Esteban et al. 2011), depression (Barone 2011) and anxiety (Prediger et al. 2012).

Another hallmark of PD pathophysiology is the development of oxidative stress. In the present study, a significant increase in lipid peroxidation was recorded in the striatum and midbrain of reserpine-induced PD animals, accompanied by a significant decrease in NO and GSH levels. Dopaminergic neurons in the SNc are especially sensitive to oxidative stress due to their reduced antioxidant capacity, increased iron concentration (Ben-Shachar et al. 1995) and the abundance of DA which is susceptible to oxidative deamination by monoamine oxidases (MAO) resulting in H2O2 production (Sherer et al. 2003). Blocking the vesicular uptake of DA leads to the accumulation of neurotoxic DA oxidation products (Caudle et al. 2008). H_2_O_2_ reacts with iron through the Fenton reaction and produce reactive and destructive hydroxyl radicals causing lipid peroxidation, amino acids modification and DNA mutations (Sherer et al. 2003). DA also reacts with molecular oxygen to give dopamine-quinones which deplete glutathione, producing reactive oxygen species (ROS) in this process (Tsang and Chung 2009).

Glutathione is an intracellular antioxidant which plays a crucial role in maintaining the intracellular redox balance and protecting neurons from oxidative damage (Circu and Aw 2008). Glutathione acts as a substrate for glutathione peroxidase and glutathione-S-transferases, and has a direct free-radical scavenging ability. The reduction in GSH level, in the present study, indicates its consumption in its attempt to attenuate the oxidative stress induced by reserpine.

Related to the induced oxidative stress, a significant reduction in NO levels occurred in the present PD model. This does not coincide with other studies that reported increased NO levels in reserpine-treated rats (Bilska et al. 2007; Chen et al. 2016) which lead to the formation of reactive nitrogen species, and nitrosative stress in PD (Olanow 2007). However, the reduced levels of NO, in the current study, may result from the reaction of NO with superoxide anion to produce a more powerful oxidant, peroxynitrite (ONOO^−^) that could cause more intense damage to cellular components such as lipids, proteins and DNA (Calabrese et al. 2007). The present neurochemical, behavioral and histopathological results confirm the reserpine-induced PD animal model, used in the study.

Each of cerebrolysin and lithium attenuated the decrease in DA content in the striatum of reserpinized rats while only lithium restored midbrain DA levels to control values. An improvement in DA levels was clear after the administration of cerebrolysin, alone or combined with lithium but failed to restore the neurotransmitter levels to control values. These neurochemical effects were accompanied by increased latency to grip loss which indicates the ability of reserpinized animals treated with cerebrolysin, lithium or their combination to hold onto objects. The restoration of striatal DA and improvement of midbrain DA after cerebrolysin, lithium and their combination may underlie the increase in grip strength as they relieve the inhibition of the thalamus and thus restore the corticospinal muscle activation. An improvement in the histological profile was also prominent after the three treatments.

The effect of cerebrolysin on dopamine levels agrees with previous studies which reported that cerebrolysin restored the dopaminergic content in rotenone-induced (Abdel-Salam et al. 2014) and 6-hydroxydopamine-induced (Noor et al. 2016) models of Parkinson’s disease and reserpine-induced model of depression (El-Marasy et al., 2021). It has been reported that cerebrolysin prevented the depletion in TH-immunoreactivity in a PD model (Abdel-Salam et al. 2014) and enhanced neurogenesis in the ischemic brain (Rockenstein et al. 2003). Thus, the increase in DA levels in the striatum and midbrain of the present reserpine-induced PD model after cerebrolysin may be attributed to the protective and neurotrophic effects of the drug.

On the other hand, Carlsson and Dixon (2018) reported that lithium treatment attenuated the deficits in dopamine neurotransmission and increased the occurrence of synaptic proteins which are important for DA signaling after traumatic brain injury (TBI). Can et al. (2016) suggested that lithium treatment affects DA synthesis or storage, and modifies DA release during abnormally high neuronal stimulation. The present increase in DA after lithium administration reflects the ability of lithium to rectify the deficits in dopaminergic transmission resulting from reserpine. The reduction in the ameliorating effect of lithium on DA levels when combined with cerebrolysin suggests that cerebrolysin slows down the activity of lithium on monoaminergic neurotransmission as the same effects were obtained in NE and 5-HT in both areas after treatment of the reserpine-induced model of PD with each of cerebrolysin, lithium or their combination.

The present study revealed that NE and 5-HT levels were improved in the striatum of reserpine model after treatment with cerebrolysin, alone or combined with lithium. Only lithium administration restored the two monoamines to control values. Similarly, an improvement in midbrain 5-HT was obtained after cerebrolysin whereas lithium alone or combined with cerebrolysin succeeded in returning 5-HT to control levels.

It has been found that lithium affects serotonergic activity (Teo et al. 2020). Several studies have shown that lithium modulates serotonergic transmission and raises extracellular serotonin levels in the brain (Wegener et al. 2003; Kitaichi et al. 2006). In addition, chronic LiCl treatment was reported to upregulate serotonergic genes in raphe nuclei in the midbrain (Smagin et al. 2021; Brunton et al. 2011) found that lithium decreases norepinephrine release and increases serotonin synthesis. It has been reported that cerebrolysin increased hippocampal 5-HT in rats suffering from memory impairment induced by diabetes mellitus (Nassar et al. 2013).

In the present study, it was found that cerebrolysin attenuated the increase in lipid peroxidation induced by reserpine in the striatum and improved midbrain MDA and both striatal and midbrain GSH levels. This is expected in view of the reported antioxidant activity of cerebrolysin (Noor et al. 2016). The oral administration of lithium ameliorated the increase in lipid peroxidation in the striatum and midbrain of the present PD model and restored GSH levels in the two areas to nearly control values. Several studies suggested that chronic lithium treatment has a neuroprotective activity against oxidative stress (King and Jope 2005; Lai et al. 2006). In vitro, chronic treatment with lithium increased GSH levels and decreased lipid peroxidation (Nciri et al. 2013; Gawlik-Kotelnicka et al. 2016). Furthermore, lithium was shown to protect the mitochondrial electron transport chain complexes from injury induced by d-amphetamine (Valvassori et al. 2010). Considering that ROS are mainly formed in mitochondria (Callaly et al. 2015), it could be suggested that lithium produces its antioxidant effects, in the present study, by modulating the mitochondrial respiratory chain activity to reduce ROS production and enhance the antioxidant mechanisms.

Cerebrolysin inhibits brain nitric oxide (Abdel-Salam et al. 2014; Ozkizilcik et al. 2019) found that the immunoreactivity for iNOS was significantly reduced by cerebrolysin in the cortex and striatum. Nahman et al. (2012) observed that lithium decreased inducible nitric oxide synthase (iNOS) expression. Thus, the present reduction in NO levels after treatment of the reserpine-induced PD model with cerebrolysin, lithium or their combination could be explained by the decrease in iNOS expression and hence NO generation.

In the present study, a significant decrease in AChE activity was recorded in the striatum and midbrain of the reserpine-induced model of PD. The presence of acetylcholine and dopamine in abundance in the striatum emphasizes the critical roles of these neurotransmitters in the functions of the basal ganglia (Rizzi and Tan 2017). AChE is responsible for acetylcholine hydrolysis in nerve terminals to terminate its action on the postsynaptic membrane. The reduced AChE enzyme activity, in a condition of reduced dopaminergic transmission, results in an imbalance between dopamine and Ach in favor of Ach which causes hyperactivity due to continuous stimulation without degradation, thus precipitating the motor symptoms of PD. Motor-impairment is the main feature characterizing the neuropathology of PD disease (Rakha et al. 2018).

Therefore, the recorded decrease in striatal and midbrain AChE activity in the present study could be due to its consumption to overcome the increased cholinergic activity in the two areas.

The present data revealed that treatment with cerebrolysin or lithium improved the enzyme activity in both areas while their combination showed a clear synergistic effect restoring the enzyme activity to control values.

The ameliorating effect of cerebrolysin and lithium on AChE activity and dopamine may be considered an important beneficial therapeutic effect which may restore the reduced balance between dopamine and acetylcholine and explain the increased latency to grip observed in the present behavioral test. It was found that cerebrolysin reversed lipopolysaccharide (LPS)-induced decline of brain acetylcholinesterase activities (Abdel-Salam 2013). Similarly, lithium normalized the decreased AChE activity in the striatum of rats after shock stress (Geoffroy et al. 1991; Jing et al. 2013) suggested that adjustment of synaptic AChE protein stability may be one of the main targets of lithium therapy.

Several other mechanisms have been implicated to participate in PD pathogenesis including neuroinflammation and alterations in transcription factors. The present study revealed a significant increase in TNF-α and a significant decrease in NF-k in the midbrain of rat model of PD induced by reserpine. Conversely, a nonsignificant increase in NF-k was evident in the striatum of reserpinized rats accompanied by a nonsignificant decrease in TNF-α.

It has been reported that upregulation of interleukin 1 beta and TNF-α, two proinflammatory cytokines, occurs in the substantia nigra of PD patients and 1-methyl-4-phenyl-1,2,3,6-tetrahydropyridine (MPTP)-treated mice (Chung et al. 2010; Lofrumento et al. 2011). The chronic upregulation of TNF-α expression causes a gradual loss of DA neurons in the SN and their striatal terminals and recruitment of monocytes and macrophages (Chertoff et al. 2011).

It is clear that the present increased pro-inflammatory cytokine TNF-α induces progressive loss in midbrain dopaminergic neurons after reserpine. This in turn results in the generation of ROS which aggravate the neuronal damage as observed in the present histopathological data. Consistent with the present conclusion, increased TNF mRNA and protein were detectable in the midbrain in animal models of PD as 6-hydroxydopamine (Xu et al. 2022), MPTP (Ferger et al. 2004), and LPS (Gao et al. 2002). In this context, reserpine has been reported to cause elevation of brain IL-1β expression and IL-1β which binds to IL-1 receptors and generates ROS, which then activate the NF-κB signaling pathway (Huang et al. 2004). The reserpine –induced PD model in this study showed elevated levels of midbrain TNF-α which indicate an inflammatory response, which could probably be mediated by the state of oxidative stress.

Nuclear factor-κB regulates the activation of inflammatory responses in both astrocytes and microglia and thus the production of proinflammatory molecules, such as tumor necrosis factor (TNF- α) and inducible nitric oxide synthase (iNOS) (Glass et al. 2010) and oxidative stress (Pang et al. 2019). Specifically, oxidative stress can stimulate NF-κB-induced neuroprotective signaling (Lingappan 2018), which may repress autophagy and autophagy-dependent apoptosis (Nandy et al. 2018). Another evidence for the involvement of NF-κB in the control of neuronal responses to dopaminergic transmission is the presence of two sites for NF-κB within the DA D2 receptor regulatory region (Bontempi et al. 2007). NF-κB dysregulation has been shown to contribute to the neurodegenerative mechanisms underlying PD (Hunot et al. 1997). Thus, the reduced levels of NF-kappa in the present study in the midbrain of reserpine model of PD may be attributed to its dysregulation due to the high levels of TNF-α and oxidative stress.

In the present study, the treatment of reserpine model with cerebrolysin resulted in an improvement in both midbrain TNF and NF-kappa which coincided with the improvement in oxidative stress parameters after cerebrolysin. Lithium exhibits anti-inflammatory and antioxidant properties (Rosenblat et al. 2014; Data-Franco et al. 2017). Nonetheless, Bristot et al. (2019) found that the therapeutic concentration of lithium did not affect the levels of cytokines after lisdexamfetaminedimesylate challenge. Nahman et al. (2012) observed that lithium decreased the expression of iNOS and secretion of TNF-α only after an extra-therapeutic dose of the drug. In the present study, the ability of lithium to restore NF-k may be mediated by its antioxidant activity. Its inability to counteract the increase in TNF may be due to the low dose of lithium employed. NF-kappa B regulates numerous physiological functions and can be either protective or noxious.

The present study provides evidence for the potential therapeutic effect of both cerebrolysin and lithium, alone or in combination, on the reserpine-induced PD animal model.

It may be concluded that cerebrolysin and/or lithium showed promising therapeutic potential against the variations induced in the present reserpine model of PD. This effect was mediated by their amelioration of the changes in dopaminergic transmission induced by reserpine. It was clear that the antioxidant and anti-inflammatory effects of both drugs played a significant role in their therapeutic potency. However, the ameliorating effects of lithium on the neurochemical, histopathological and behavioral alterations induced by reserpine were more prominent than those of cerebrolysin alone or combined with lithium. This was evident in oxidative stress parameters and NF- Kappa, suggesting that the combination of both drugs was not always beneficial. However, a limitation in the present study was the use of single doses for both drugs and a single behavioral test. Moreover, the time period could have been extended. Therefore, further studies are recommended to investigate the potency of the combination of different dosage regimens of the two drugs to produce the highest efficacy in PD treatment.

## Data Availability

All data are available on request.

## Abbreviations

AChE: Acetylcholinesterase
ANOVA: one-way analysis of variance
PD: Parkinson’s disease
CBL: Cerebrolysin
CNS: Central Nervous System
CU-IACUC: Cairo University - Institutional Animal Care and Use Committee
DA: Dopamine
DTNB: 5,5’-dithiobis-(2-nitrobenzoic acid)
EDTA: Ethylenediaminetetraacetic acid
GSH: Reduced glutathione
H&E: Hematoxylin–eosin
5-HT: Serotonin
iNOS: Inducible nitric oxide synthase
i.p.: Intraperitoneal.
MAO: Monoamine oxidases
MDA: Malondialdehyde
MPTP: 1-methyl-4- phenyl-1,2,3,6- tetrahydropyridine
NE: Norepinephrine
NED: N-(1Naphthyl) ethylenediaminedihydrochloride
NF-κ: Nuclear factor kappa B
NO: Nitric oxide
ONOO^−^: Peroxynitrite
OPT: O-phthalaldehyde.
ROS: Reactive oxygen species
S.E.M.: Standard error of the mean
SNc: Substantia nigra pars compacta
TBA: Thiobarbituric acid
TBI: Traumatic brain injury
TCA: Trichloroacetic acid
TNF-alpha: Tumor necrosis factor-alpha
VMAT2: Vesicular monoamine transporter 2
